# P-551. The Effects of Antiretroviral Therapy on Immune Activation, Inflammation, Cellular Changes and Clinical Outcomes in Elite Controllers

**DOI:** 10.1093/ofid/ofae631.750

**Published:** 2025-01-29

**Authors:** Elizabeth Chan, Lei Zhou, Brinda Emu, Elijah Paintsil, Lydia A Barakat

**Affiliations:** Yale School of Medicine, Sharon, Massachusetts; BOSTON UNIVERSITY, Boston, Massachusetts; Yale University, New Haven, CT; Yale University, New Haven, CT; Yale School of Medicine, Sharon, Massachusetts

## Abstract

**Background:**

Elite controllers are people living with HIV (PLWH) who maintain their HIV viral load (VL) < 50 copies/mL without antiretroviral therapy (ART). Viremic controllers are persons who have HIV who are able to keep their VL < 2000 copies/mL without ART. Current published data does not clearly define the benefits of ART nor the adverse effects of ART in elite controllers. Evaluation of the effects of ART on immune activation, inflammatory markers, mitochondrial and cellular function in elite controllers can better inform utilization of ART in this group.

**Figure:**
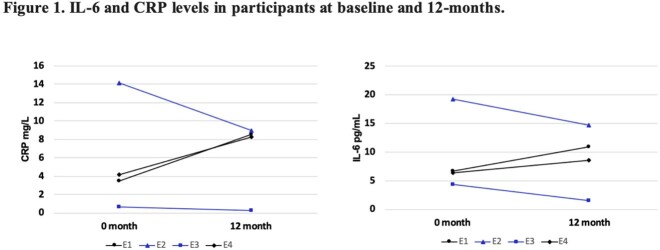

**Methods:**

Four PLWH—3 elite controllers and 1 viremic controller— were enrolled from our HIV academic center clinic. Participants were women aged 45 to 60 years old who were diagnosed with HIV for 5 to 35 years. After shared decision making with their primary care provider, 2 of the 4 participants were started on ART, one on Triumeq and the other on Cabenuva. Participants were followed from 0 to 12 months. At each visit, routine standard of care labs, as well as inflammatory lab markers IL-6 and CRP were drawn. We assessed their mitochondrial function measuring mitochondrial membrane potential, mitochondrial DNA (mtDNA) deletions, apoptosis, and reactive oxygen species (ROS) production.

**Figure:**
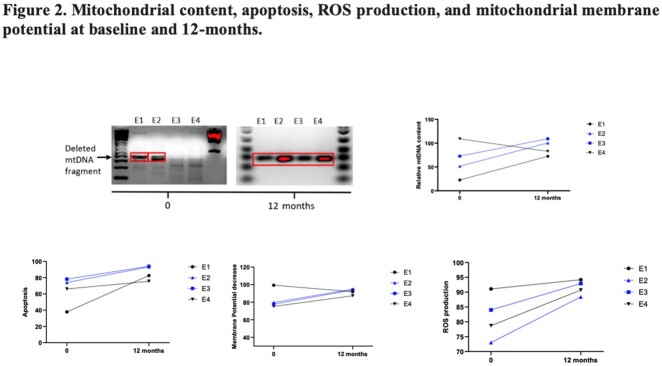

**Results:**

Compared to baseline levels, inflammatory markers IL-6 and CRP both decreased at 12-months in participants started on ART (E2 and E3), whereas these markers increased in controls (E1 and E4) (Figure 1). All participants have increased apoptosis and ROS production at 12-months compared to baseline (Figure 2). At the 12-months visit, participants in the ART group (E2 and E3) showed elevated levels of mtDNA deletion, apoptosis, ROS production, and a reduction in mitochondrial membrane potential (Figure 2).

**Conclusion:**

From our small cohort, the observed IL-6 and CRP decrease suggest decreased inflammation at one year after elite controllers start ART. However, decreased mitochondrial function was also noted in those on ART, which suggests possible early signs of ART toxicity. This preliminary data emphasizes the need for further studies to better understand the risk-benefit of initiating ART in elite controllers, including markers for cellular inflammatory markers.

**Disclosures:**

**Brinda Emu, MD**, Genentech/Roche: Advisor/Consultant|Theratechologies: Advisor/Consultant

